# Remote, tablet-based assessment of gaze following: a nationwide infant twin study

**DOI:** 10.3389/fpsyg.2023.1223267

**Published:** 2023-10-03

**Authors:** Frederick Shic, Kelsey Jackson Dommer, Jessica Benton, Beibin Li, James C. Snider, Par Nyström, Terje Falck-Ytter

**Affiliations:** ^1^Center for Child Health, Behavior and Development, Seattle Children's Research Institute, Seattle, WA, United States; ^2^Department of Pediatrics, University of Washington School of Medicine, Seattle, WA, United States; ^3^School of Computer Science and Engineering, University of Washington, Seattle, WA, United States; ^4^Uppsala Child and Babylab, Department of Psychology, Uppsala University, Uppsala, Sweden; ^5^Development and Neurodiversity Lab, Department of Psychology, Uppsala University, Uppsala, Sweden; ^6^Center of Neurodevelopmental Disorders (KIND), Karolinska Institutet, Stockholm, Sweden; ^7^Swedish Collegium for Advanced Study (SCAS), Uppsala, Sweden

**Keywords:** eye-tracking (ET), remote assessment, twins, gaze following, infant development, WebCam applications

## Abstract

**Introduction:**

Much of our understanding of infant psychological development relies on an in-person, laboratory-based assessment. This limits research generalizability, scalability, and equity in access. One solution is the development of new, remotely deployed assessment tools that do not require real-time experimenter supervision.

**Methods:**

The current nationwide (Sweden) infant twin study assessed participants remotely via their caregiver's tablets (*N* = 104, ages 3 to 17 months). To anchor our findings in previous research, we used a gaze-following task where experimental and age effects are well established.

**Results:**

Closely mimicking results from conventional eye tracking, we found that a full head movement elicited more gaze following than isolated eye movements. Furthermore, predictably, we found that older infants followed gaze more frequently than younger infants. Finally, while we found no indication of genetic contributions to gaze-following accuracy, the latency to disengage from the gaze cue and orient toward a target was significantly more similar in monozygotic twins than in dizygotic twins, an indicative of heritability.

**Discussion:**

Together, these results highlight the potential of remote assessment of infants' psychological development, which can improve generalizability, inclusion, and scalability in developmental research.

## Introduction

Currently, researchers interested in the psychological development of infants generally have two options: either to rely on remote but indirect assessment (e.g., caregiver surveys) or to invite families into their laboratories to conduct their experiments and assessments. While the first option is feasible for many types of questions, there are limits to what one can reliably assess via parent reports. Therefore, over the last decades, more and more “Babylabs” have emerged, often with highly specialized equipment for the measurement of infant behavior and brain development. These laboratories currently produce a substantial part of what we consider knowledge about infant development. The advantages of this approach include standardized measurement by trained personnel, advanced methods and techniques tailored to research questions, and the possibility to communicate directly with parents about the research and address any concerns that may emerge.

However, laboratory-based infant development research has a number of serious limitations as well. First, the approach typically leads to small and/or unrepresentative samples, limiting statistical power and generalizability. In Sweden, for example, there are a handful of dedicated “Babylabs,” all located in major university cities. In other words, large parts of the country (e.g., rural areas) are effectively excluded from participation in infant research. This is problematic because the population of the bigger university cities is not representative of the country's overall population (e.g., in terms of socioeconomic status). Second, the unfamiliar situation and equipment in the laboratory may induce stress or uncertainty in parents and infants, limiting the ecological validity of the research. Third, the in-laboratory approach limits the type of research questions one can address (e.g., due to limited sample size or restricted participant characteristics). Relatedly, the results of in-laboratory assessments often have unknown applied utility (e.g., in terms of screening for behavioral problems) because these assessments may not resemble standards that can be deployed easily or practically in communities, homes, or clinics. Fourth, the lack of standardization reduces the options for reproducing studies conducted by independent groups. Finally, in-laboratory-based testing is typically associated with very high costs per tested infant.

Against this backdrop, we developed a tablet-based experiment of infant gaze behavior and attention to be deployed asynchronously (without real-time experimenter supervision) in the homes of families recruited from all over Sweden via population registers. We chose to focus on gaze for three reasons: (1) Infants learn and communicate via their gaze behavior: even before they can walk or crawl, they use their gaze to select what to attend to and what to ignore in their environment; (2) eye tracking is a well-established and frequently-used technique in laboratory-based infant research, allowing us to link our findings to previously established results; and (3) if successful, our approach could offer an alternative, highly scalable methodology for indexing infant attention.

More specifically, we chose to assess gaze following (the tendency to follow another person's gaze). Gaze following is a theoretically and clinically important behavior relevant to the construct of “joint attention” and is believed to be key for infant socio-communicative development (Carpenter et al., 1998). Further, it is well-studied, meaning that we could anchor our results in a pre-existing knowledge base. It is assessed using a straightforward experimental design with clear operational definitions that allows for both simple (manual) and, in the future, more advanced (automatic and computer-vision-based) behavioral coding options. The gaze following task used in this study shows an actress in the center of the screen flanked by two objects. What is measured is whether and how fast the infant follows the actress's gaze when she directs her attention to either of the objects on either side. Previous research using conventional eye tracking has shown that gaze following develops rapidly over the first and second years of life (Moore, 2008; Del Bianco, 2019). The physical layout of established gaze following paradigms, with a central point of origin and clear movement required toward peripheral targets, lends itself very well to manual coding and, in the future, computer-vision-based coding. Our current study, using manual coding, takes advantage of this layout and expects accuracy on par with the decades of studies that have used manual coding of peripheral looking (Jongerius et al., 2020). Furthermore, gaze following is facilitated by clear cues, such as when the eyes and head move together toward the target (Moore and Corkum, 1998). Together, these previous findings provide clear predictions about what we expect to observe in our tablet-based approach. The task also allowed us to test a further theoretically motivated (but less empirically validated) question of whether gaze following depends on situational factors (e.g., the presence of a clear communicative context). Some research has indicated that infants only follow gaze after having first heard cues such as infant-directed speech, while other, more recent studies indicate that these results may reflect attention/arousal in general rather than highly specific communicative cues (Senju and Csibra, 2008; Ishikawa and Itakura, 2019).

In terms of the target population, we chose to recruit infant twins. The reason for this was that we wanted to showcase the fact that we, with this approach, can reach groups who are typically not studied in infant laboratories. Furthermore, to our knowledge, no study has assessed the influence of genetics vs. environment on gaze following and joint attention in young children or infants. In other words, studying twin populations allows us to address the question: Do individual differences in gaze following reflect differences in genetic makeup, environments, or both? With twin data, the total variance in a trait can be divided into common environmental variance, genetic variance, and non-shared environmental variance (the last also incorporates measurement error). It is assumed that all twins who are raised together (both MZ and DZ) share common environmental influences. Identical (monozygotic; MZ) twins share 100% of their segregating DNA, and fraternal (dizygotic; DZ) twins share, on average, 50% of their segregating DNA. Thus, differences between twins in any given MZ twin pair can be attributed to non-shared environmental variance only, whereas for DZ twin pairs, variance is attributable to both non-shared environmental and genetic variance. Assuming that environmental factors are similar for DZ and MZ twins, on average, a pattern of higher within-pair similarity for MZ than DZ twins is assumed to primarily reflect genetic influences on the trait (Polderman et al., 2015).

Although the idea of remote assessment of gaze behavior in the homes of families is not new, surprisingly few results have been published to date. At the time of writing of this manuscript, different ideas and technical platforms were being discussed and tested around the world. In particular, currently, the ManyBabies-AtHome (MBAH) project has been evaluating the platform Lookit (Scott and Schulz, 2017) to collect preferential-looking data remotely from infants (Zaadnoordijk et al., 2021). This platform for remote gaze tracking using video recordings from tablets showed promising results in children aged 1 year or older in one validation study (Scott et al., 2017).

Taken together, this study aimed to develop, employ, and evaluate new technology for at-home eye-tracking data collection in young infants. The specific aims were to

1. Develop internet-based technology for remote, in-home assessment of infant attention, administered by the parents without real-time researcher involvement.
2. Assess infants' gaze following performance with this technology using a task for which performance could be compared to known developmental and experimental effects obtained by previous in-laboratory studies. specifically, we predicted (1) that we would see a sharp increase in gaze-following ability over the age range examined (3-17 months) and (2) that the stimuli combining eye movements and head turns toward a peripheral target would elicit more gaze following than stimuli where only the actress's eyes moved toward the target (Nyström et al., 2019, biological psychiatry). Regarding the impact of infant-directed speech, we had no directional hypothesis, given the mixed findings in previous research (Senju and Csibra, 2008; Ishikawa and Itakura, 2019).
3. Evaluate the approach's suitability for special infant populations, which can be difficult to recruit in high numbers (e.g., due to geographical spread). As noted above, we recruited infant twins. Although our study turned out to be underpowered for formal twin modeling, we could include some preliminary data on the potential genetic contribution to gaze following that can be followed up in future research.

## Methods and materials

### Overview

We developed an iOS iPad application (the Karolinska Infant iPad Twin study application or “KiiTs”) that records a video of a participant's face time locked to the video stimulus presentation. We deployed the tablet-based application remotely to infant twins throughout Sweden to examine developmental and genetic factors associated with gaze-following behaviors in response to classical eye and face movement cues. The study protocol was approved by the regional ethics review board in Stockholm, which also ensured that informed consent was obtained from all participating families.

### Participants

Throughout 2019, based on the Swedish Population Registry, we sent out recruitment letters to a total of 1,597 families with twins aged between 3 and 20 months (this registry includes information about the date of birth and gender). All twin families in the target area in the period in question were invited, except for families living in the greater Stockholm area (due to participation in another twin study; see recruitment map, Supplementary Figure S1).

The recruitment letter specified that only parents whose children were born in or after gestational age week 34 were eligible for participation (this was subsequently confirmed via a parental report in the questionnaire). Another inclusion requirement was the availability of an iPad (Apple Inc.) for participation in video data collection using KiiTs. Additionally, the recruitment letter provided clear, step-by-step instructions about the study procedures, including a picture indicating how parents/caregivers should position themselves and their children in front of the tablet (Supplementary Figure S2), general information about the study, and the research team's contact information in case families wanted additional information, had questions, or needed technical support.

The online questionnaires covered basic demographic information and basic family medical history, along with a short individual medical history for each child in the twin pair. Furthermore, parents answered questions about twin similarity, which we used to classify each pair as either MZ or DZ, using a machine learning-based approach that we have shown to have high accuracy compared with DNA-based zygosity testing in infancy (Hardiansyah et al., 2021).

Sixty-one families (122 children) provided online questionnaire data. Fifty-seven of these families (106 children) also provided video data via the application. One family was excluded due to severe twin-to-twin transfusion syndrome (reported via the parent questionnaire); otherwise, no exclusions were made based on family characteristics. None of the infants had a parent-reported diagnosis related to development. Of the 56 families, 8 families uploaded video data for only one infant; the remaining families uploaded video data for both infants. Sample characteristics of the included families (56 families; 104 children) are provided in Table 1. As compared to a contemporaneous study of infant twins localized to a single, medium-sized metropolitan area in the same country (Babytwins Study Sweden (BATSS); Falck-Ytter et al., 2021), the sample of this study included families with lower salaries (this study's Salary Index (range 1-10): M = 5.71, SD = 2.28; BATSS: M = 6.57, SD = 2.32; and *t*(357) = 2.40, *p* = 0.017) but similar levels of education (this study's Education Index (range 1-4): M = 3.31, SD = 1.14; BATSS: M = 3.29, SD = 0.85; *t*(365) = 2.80, *p* = 0.005). For details on the Salary Index and Education Index, see Falck-Ytter et al. (2021) and the table legend of Table 1.

**Table 1:** Family characteristics.

**Family characteristics**	**Values**
Number of families	*N =* 56
Number of children	*N =* 104
Twin ages (months)	M = 8.6, SD = 3.6 months, Range = [3.4, 16.9]
Twin couplet Zygosity	Monozygotic (MZ) *N =* 27 (51.8%) Dizygotic (DZ) *N =* 21 (41.1%) Unknown *N =* 4 (7.1%)
Twin gender assigned at birth	Both Boys *N =* 18 (32.1%) Both Girls *N =* 28 (50.0%) One Boy, One Girl *N =* 10 (17.9%)
Twin gestational age (weeks)	M = 36.8, SD = 1.5 weeks
Respondent	Mother *N =* 50 (89.3%) Father *N =* 1 (1.8%) Unknown *N =* 5 (8.9%)
Mother's age (years)	M = 32.1, SD = 4.9 years, Range = [22.3, 47.8]
Father's age (years)	M = 34.6, SD = 7.2 years, Range = [24.4, 67.2]
Family income^1^	M = 5.71, SD = 2.28 years, Range = [1, 10]
Parent education^2^	M = 3.31, SD = 1.14, Range = [1, 10]

^1^Family income per month. Scale 1 to 10 where 1 = <20K, 2 = 20-30K, 3 = 30-40K, 4 = 40-50K, 5 = 50-60K, 6 = 60-70K, 7 = 70-80K, 8 = 80-90K, 9 = 90-100K, and 10 = >100K (SEK). 1.0 SEK = 0.105 $ (USD), 100K SEK/Month = $126K USD/Year at the time of the study. ^2^Education level on a scale from 1 to 4, where 1 = Primary, 2 = Secondary, 3 = Undergraduate ( ≤ 3 years) and 4 = Postgraduate level (>3 years).

Families who participated received a gift voucher of ~€ 10, sent by regular mail. The study was approved by the Ethics Committee in Stockholm, and parents provided written consent (obtained within the application).

### Tablet app/process development

The KiiTS app (see Supplementary Figure S3 for app screenshots and flow) was developed to be compatible with Apple's App Store requirements and to meet the GDPR data security requirements (GDPR, 2022). The app allowed for the collection of remote consent from a parent, followed by an opportunity to record one video session per participating twin. Families were given specific access to the app using a code they received in their physical letter in the mail to ensure only families included in our population of interest could participate. Each child was also given a Child ID (either A or B) to ensure that we could tie questionnaire answers to the correct eye-tracking recording. The app itself collected information that the family entered, such as signature and date or Family ID, as well as limited meta-data to allow for the analysis of data loss and study dropout rates. The app deployed identical experimental stimuli across all participants. During the experimental stimuli, the app recorded a video of the child watching using the front-facing iPad camera, but it did not record any audio information. The video was recorded at a rate of 30 frames per second. It was sent along with a log file that provided timing information on exactly which stimuli were being shown at what time point in the recorded video. These data were transferred securely via Box to the data analysis team at Seattle Children's Research Institute (SCRI). This allowed the coding team at SCRI to precisely time lock manual coding of eye-gaze behavior with the stimuli being presented.

### Data collection session

After an in-app consent form was signed, the parent user was presented with instructions to seat the child securely in front of the iPad in a well-lit room. It was requested that the iPad be stable and vertical on a table (i.e., not be held by the parent) and that the lighting illuminate the face well, without any harsh shadows or backlighting. Once the parent clicked that they were ready, a screen was shown with video feedback from the front-facing camera, and the parent was asked to center the child within the frame. They then clicked to proceed and after a final gentle reminder to hold the child still, the paradigms automatically began. A data collection session started with a calibration sequence and consisted of interleaved blocks of two paradigms (Gaze Following and Social Preference Task) and additional calibrations, followed by a third paradigm (Short Term Memory Task). We will only be evaluating and discussing Gaze Following in this study.

### Experimental task

**Figure 1:**
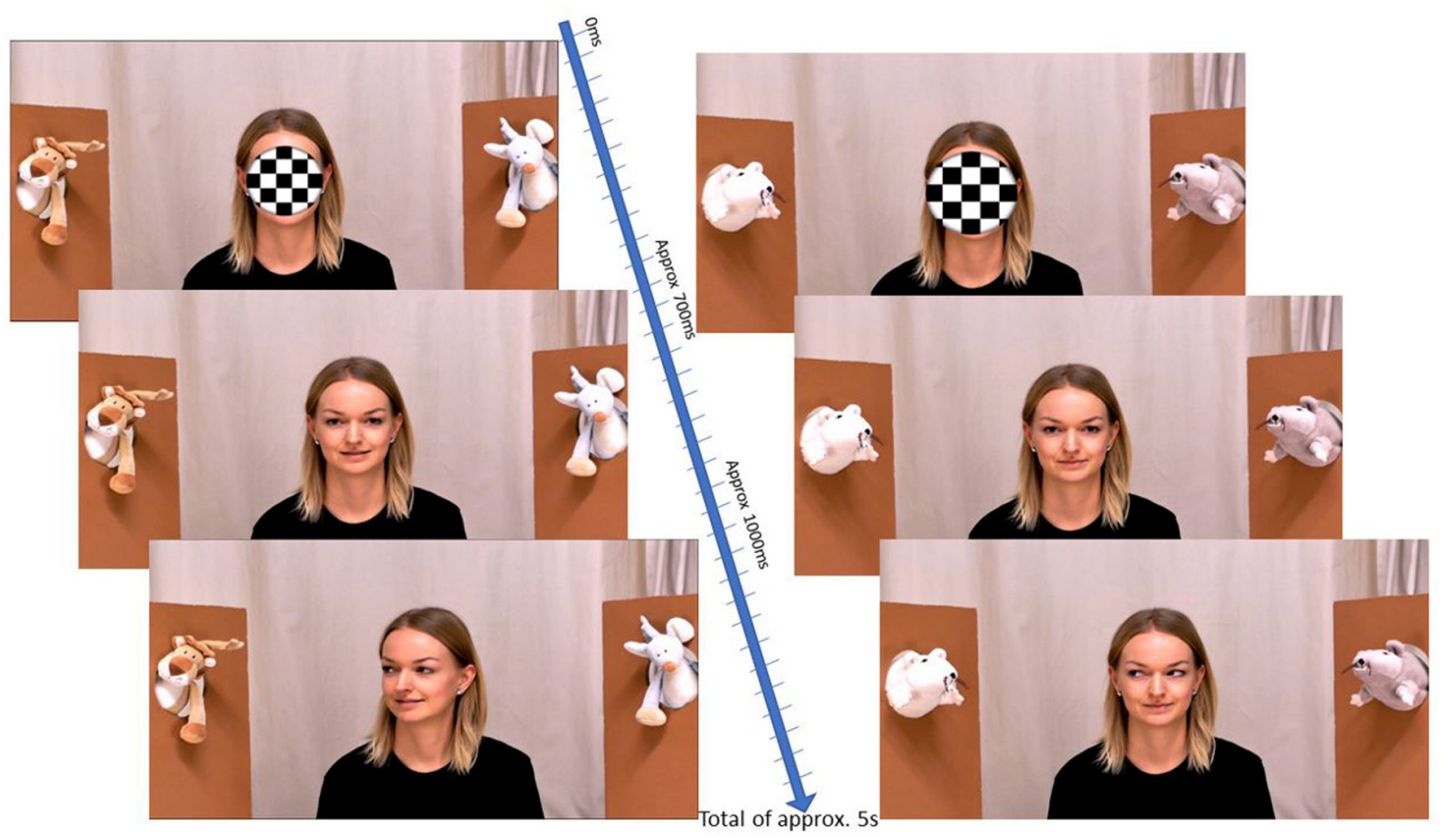
Example trial flow. Screenshot examples of the flow of a trial. The left image exemplifies the Eyes and Head condition while the right demonstrates the Eyes only condition.

Stimuli (see Figure 1) were created to match the live interaction stimuli used in Thorup et al. (2016) and Nyström et al. (2019). A total of 32 trials were administered in two-trial blocks. The trials lasted ~5 s each. The stimuli depicted an actress centered on the screen, flanked by toys on each side of her at her eye level. The trials began with an audiovisual attention grabber presented simultaneously with sound for 700 ms. The attention grabber was a looming black and white checkered circle superimposed over the actress's face that completely obscured all facial features; the audio sound (which began and ended with the attention grabber's presentation) consisted of either a friendly musical tone (Non-social Sound condition) or a woman's voice saying “hey hey” in child-directed speech (Social Sound condition). After this presentation, the attention grabber shrunk out of view, revealing the actress's face oriented toward the camera in such a manner so as to emulate direct eye contact with the viewer. The actress remained looking directly at the camera for approximately an additional 300 ms. The actress then turned either her eyes only (Eyes Only condition) or both her eyes and head (Eyes and Head condition) toward one of the two toys (which defined the target side for that trial) and froze in that position until the trial ended.

The combination of gaze cue type and pre-trial sound type created four fundamental conditions:

1. Eyes Only + Non-social Sound
2. Eyes Only + Social Sound
3. Eyes and Head + Non-social Sound
4. Eyes and Head + Social Sound

Each condition was presented an equal number of times and was balanced so that there was an equal chance of the target direction being right or left within each condition.

### Deriving results

**Figure 2:**
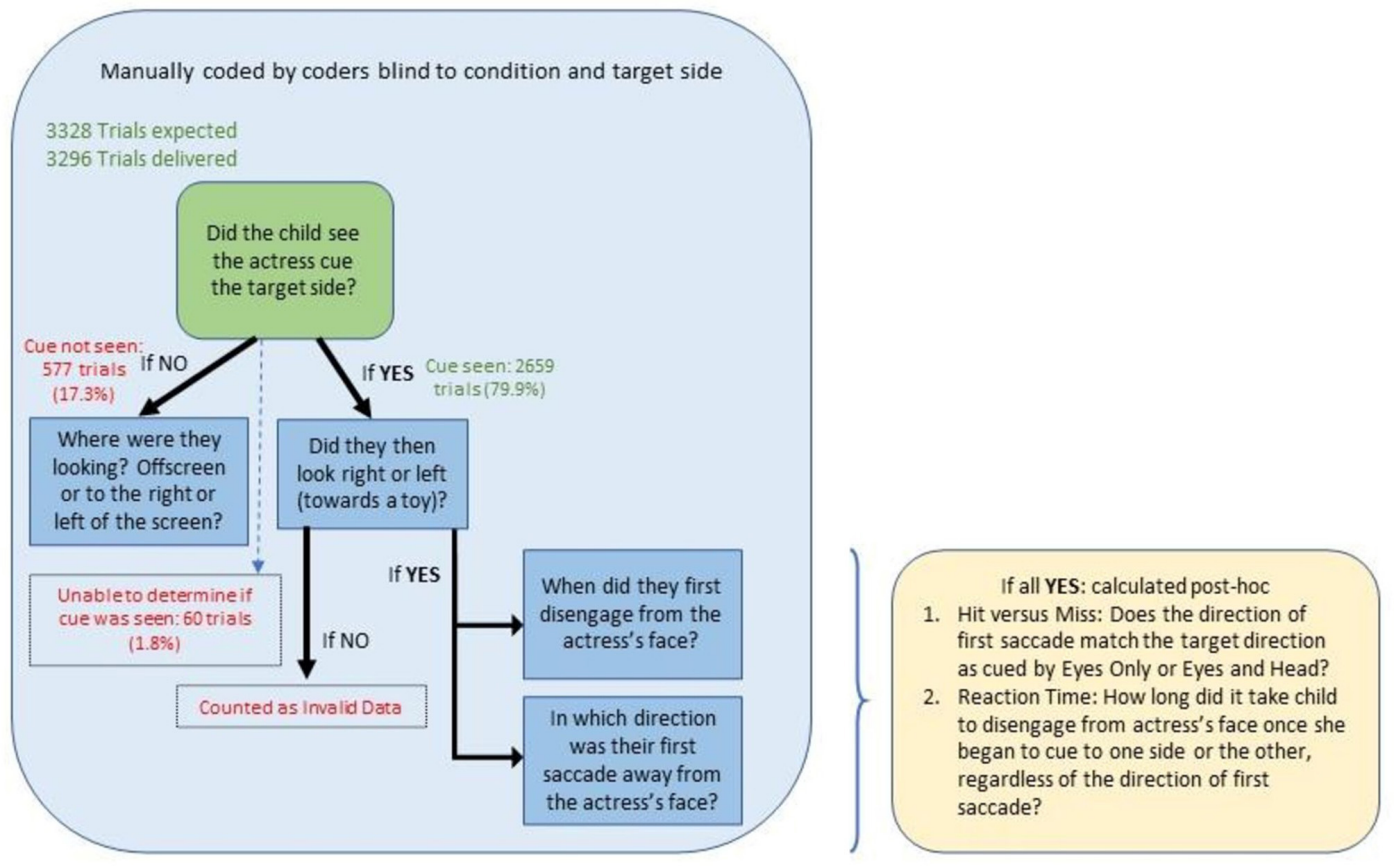
Manual coding steps. This diagram documents the general flow of manual coding and includes some basic numbers to clarify how the project arrived at its final valid dataset. The question branches indicate what was recorded by the coding team for each trial.

At the end of a data collection session, the parent user was asked to verify the upload of the files created during the session, as per GDPR guidelines. Upon receipt of those files, a study team at SCRI manually coded the videos following strict protocols. Trail validity depended on whether the child was attending to the actress's face at the start of her motion to the target side. If the child was not paying attention to the start of this cue, then the coder marked where the child was looking instead and did not continue to code that trial (see Figure 2). This trial is slightly different from the original study (Thorup et al., 2016) but was necessary due to the intensive efforts that manually coding frame-by-frame took compared to automated eye-tracking techniques. For those trials where the participant did view the start of the cue, instances of the child disengaging from the actress's face were manually coded, and the direction in which they took their first saccade was identified. The direction could be either right, left, off-screen, or n/a for those trials where they did not move away. Analyses were then conducted to determine whether the trial was a “hit” or “miss” and what the reaction time was. A hit was defined as the child watching the start of the cue and then having a first saccade in the target direction. A miss was defined as a child watching the start of the cue, followed by a saccade to the side opposite the target direction. Reaction time was defined as the amount of time from the start of the cue to the first saccade away from the center. Valid trials were those trials in which hit-or-miss and reaction times could be computed; conversely, invalid trials were those in which the child never looked at the cue or never moved or looked offscreen subsequent to looking at the cue (see Figure 2). For all manually coded variables, the coder had the option to deem it impossible to code with certainty, although this outcome was very rare. Videos were largely double-coded, and when discrepancies occurred, the outcome was discussed with a lead coder and either agreed upon by all parties or deemed impossible to code with certainty.

### Analyses

Analyses were conducted to address three primary research questions. Each analyses considered five independent variables. The age group was defined as older (> = 7.5 months, the calculated median of the sample) or younger, although a parallel analysis treating age as a continuous variable was also conducted (presented in Supplemental material, together with the rationale for considering age as a categorical variable as the primary approach). The gender of the participants was recorded as reported by the parents as either boy or girl. Zygosity was either monozygotic (MZ) or dizygotic (DZ) and was collected via parent report. The pre-trial sound type was either a Social Sound (“hey hey” during the attention grabber) or a Non-social Sound (a bell sound during the attention grabber). Finally, the gaze type was either Eyes Only or Eyes and Head. Outcome variables included rates of data loss (percentage of invalid trials), percentage of hits, and reaction time. The proportion of hits rather than the number of hits was selected as an outcome measure due to the varying number of trials per participant.

It was hypothesized that age group and sound would have independent effects on the rates of data loss such that older participants would have less data loss and a social sound would elicit less data loss. Similarly, it was posited that age group and gaze type would have independent effects on the proportion of hits: older participants were considered more likely to provide more hits than younger participants, and it was predicted that the Eyes and Head condition would scaffold gaze following and provide a greater proportion of hits than the Eyes Only condition. An exploratory analysis was performed to gauge the effect of the Social vs. Non-social Sound in the attention grabber on the hit rate. This was, to our knowledge, the first project to include this manipulation, and it was unclear if it would have an independent effect, interact with age or gaze type, or have a non-significant effect. It was also predicted that the age group would have an independent effect on reaction time so that older participants would have a faster disengagement time than younger participants.

All outcome variables were modeled using a mixed-effects model [using the “glmmTMB” package in R (Brooks et al., 2017)] that included the fixed effects of condition {i.e., gaze cue type (Eyes Only or Eyes and Head) × pre-trial sound [human voice (Social Sound) or bell sound (Non-social Sound)]} as well as age group (age <7.5 months or ≥ than 7.5 months) and gender at birth (boy or girl). Additionally, a random intercept of twin ID (twin A or twin B) was nested within the family ID. Analyses of reaction times also included fixed effects of gaze-following accuracy [hit or miss]. ANOVA was conducted using Type III Wald chi-squared tests, followed by planned contrasts of age group, gaze cue type, and pre-trial sound.

In light of the small sample size and in agreement with a recent eye-tracking study among toddlers (Constantino et al., 2017), we conducted exploratory analyses of the effects of zygosity on all outcome variables by comparing the relative strength of intraclass correlation coefficient (ICC) values between twin couplets in each group using the function *icc* in R (Gamer et al., 2012), with follow-up non-parametric analyses using the rank-order of values to verify significant findings. We also examined absolute differences in performance between infant twins within each family, comparing the mean of families based on zygosity against a bootstrap estimate of pairs of unrelated infants from different families (100,000 replications). For this analysis, alpha was set at 0.05 under a 1-sided null hypothesis that differences between related infants would be higher than those between unrelated infants.

## Results

A total of 3,328 trials across participants were planned for delivery. A total of 32 (1.0%) trials were lost due to early termination or data transfer failure. In 577 (17.3%) trials, participants were not looking at the cue when it appeared; in an additional 60 (1.8%) trials, it could not be determined whether the participant was looking at the cue. Subsequent to the cue, in 231 (6.9%) trials, participants failed to move their eyes to any side. In 411 (12.3%) trials, they looked away from the screen; and eye movement direction could not be assessed by coders in 12 (0.4%) trials. This left a total of 2,005 (60.2%) trials that were analyzable for gaze direction in response to cues. See Figure 2 for visual reference.

### Proportion of invalid trials

We examined the proportion of trials lost to inattention or task non-compliance (invalid trials) using a binomial mixed-effects model. Trials were considered valid if the child made a movement to the left or right side of the screen subsequent to seeing the gaze cue. Trials were considered invalid if the child was not looking at the screen when the gaze cue began, if the first eye movement subsequent to the presentation of the gaze cue was made off-screen, or if the child did not respond to the cue with an eye movement but instead maintained gaze at the actress. Trials for which the attended-to or not-attended-to status could not be determined, including trials lost for technical reasons, were excluded from analysis [104 of 3,328 (3.1%) trials].

**Figure 3:**
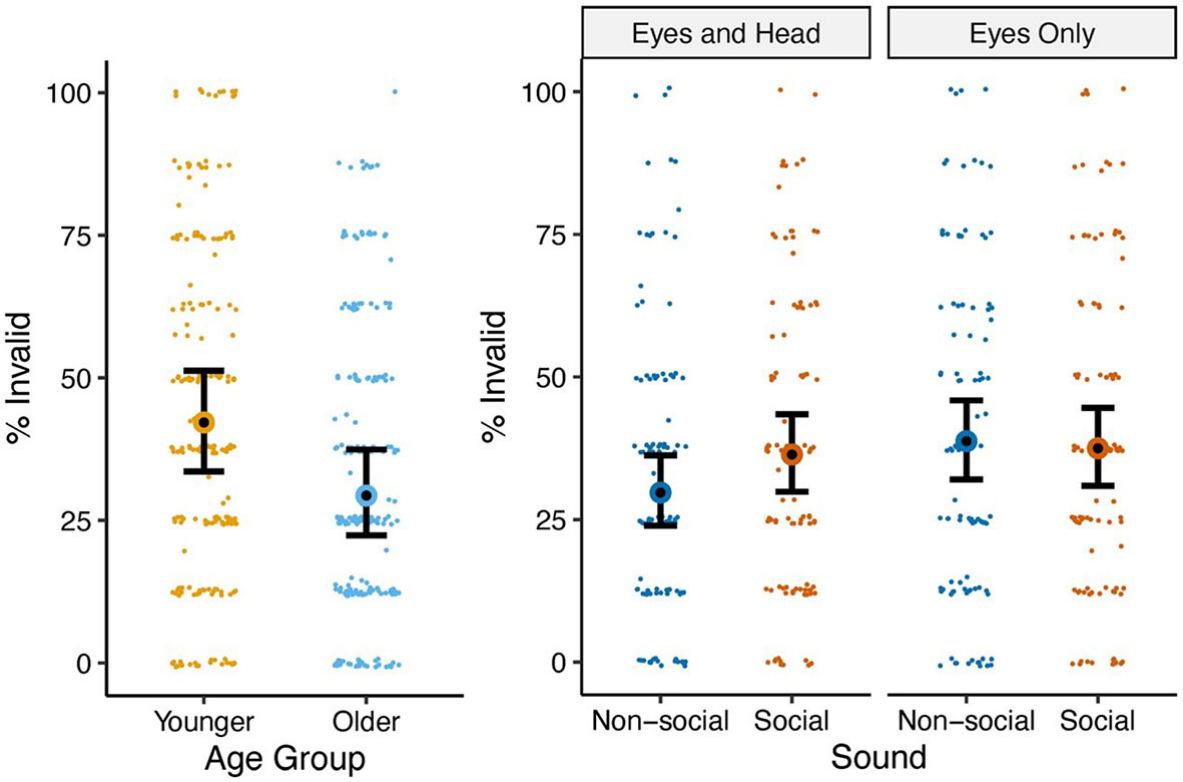
Means and standard errors of proportion of invalid trials by age group **(left)** and gaze type × sound **(right)**.

ANOVA indicated the main effects of age group and gaze cue type but not those of either gender or pre-trial sound, and it indicated a gaze cue type × pre-trial sound interaction (Table 2). Younger children incurred more invalid trials [42.2%, 95% CI (33.6%, 51.3%)] than older children [29.3% (22.4%, 37.4%)] [*t*(55) = −2.15, *p* = 0.036]. However, this effect was not replicated when age as a categorical variable was replaced by age as a continuous variable (see Supplemental material), suggesting potential non-linear behaviors or questionable strength of the detected effect. The Eyes Only condition led to more invalid trials [38.1% (32.0%, 44.7%)] than the Eyes and Head condition [33.0% (27.3%, 39.2%)] [*t*(55) = 2.75, *p* = 0.008]. The interaction effect of gaze type and pre-trial sound was driven by the lowest proportion of invalid trials occurring in the Eyes and Head + Non-social Sound condition (see Table 3 and Figure 3). All other terms were non-significant.

**Table 2:** ANOVA of proportion of invalid trials.

**Variable**	**X^2^**	***P***
Age group	4.64	0.031 ^*^
Gender	0.01	0.919
Gaze type	7.54	0.006 ^**^
Pre-trial sound	2.42	0.120
Gaze type^*^sound	4.71	0.030 ^*^

Outcome of the ANOVA for the model evaluating [Proportion of Invalid Trials ~ Age Group + Gender + Gaze Type ^*^ Pre-trial Sound + (1|Family/Child)].

**Table 3:** Estimated means of proportion of invalid trials per condition.

**Condition**	**95% CI**	**Lower bound**	**Upper bound**
Eyes Only + Non-social Sound	38.7%	32.0%	45.9%
Eyes and Head + Non-social Sound	29.7%	23.9%	36.3%
Eyes Only + Social Sound	37.5%	30.9%	44.6%
Eyes and Head + Social Sound	36.4%	29.9%	43.4%

Twin analyses did not support correlated rates of invalid data in either monozygotic [rank-based ICC (*n* = 28) = −0.026, *p* = 0.553] or dizygotic [rank-based ICC (*n* = 24) = −0.135, *p* = 0.741] twins. Similarly, bootstrap analyses did not reveal stark contrasts between sibling differences in the proportion of invalid trials for either monozygotic (average absolute difference between twins |Δ| = 20.2%, *p* = 0.088) or dizygotic (|Δ| = 21.5%, *p* = 0.163) twins as compared to unrelated-infant differences (5th percentile |Δ| = 19.1%).

### Hits vs. misses

We examined the proportion of hits (trials in which the participant was watching the start of the cue and then made their initial eye movement away from the actress's face toward the side of the screen she was looking at) relative to the total hits or misses (trials in which the participant was watching the start of the cue and then looked either toward the target or the opposite direction of the target) using a binomial mixed-effects model (similar to the approach used for the proportion of invalid trials).

**Figure 4:**
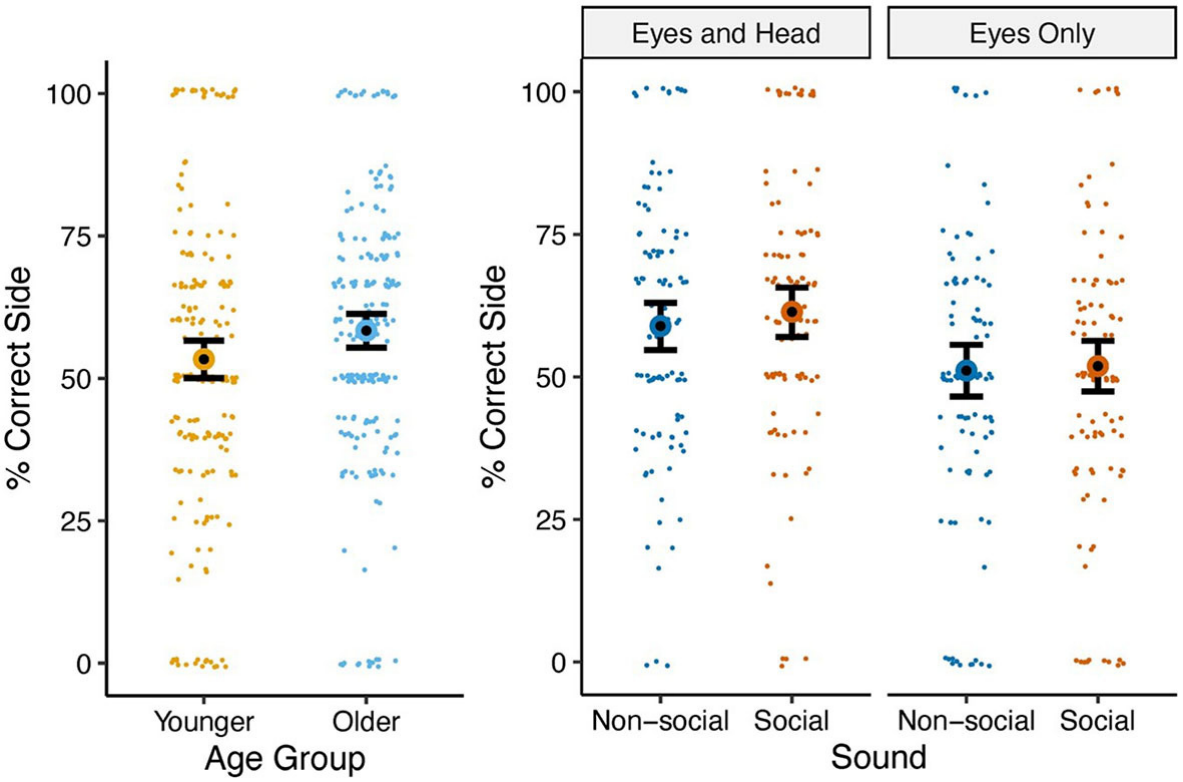
Means and standard errors of proportion of hits (i.e., “correct side” consistent with actor gaze cue direction) by age group **(left)** and gaze type × sound **(right)**.

ANOVA indicated the main effects of age group and gaze cue type, with all other terms being non-significant (Table 4). Older children had a higher probability of a hit [58.4%, 95% CI (55.4%, 61.3%)] than younger children [53.4% (50.1%, 56.6%)] [*t*(55) = 2.24, *p* = 0.029]. The Eyes Only Condition elicited a lower probability of a hit [51.5% (48.2%, 54.7%)] than the Eyes and Head condition [60.2% (57.1%, 63.2%)] [*t*(55) = −3.89, *p* <0.001) (see Figure 4 for visualization). All other terms were non-significant.

**Table 4:** ANOVA of the proportion of hits.

**Variable**	**X^2^**	***P***
Age group	5.00	0.025 ^*^
Gender	0.13	0.720
Gaze type	15.15	<0.001 ^***^
Pre-trial Sound	0.56	0.455
Gaze type^*^sound	0.16	0.690

Outcome of the ANOVA for the model evaluating [Proportion of Hits ~ Age Group + Gender + Gaze Type ^*^ Pre-trial Sound + (1|Family/Child)].

Twin analyses did not support correlated hit rates in either monozygotic [rank-based ICC (*n* = 28) = −0.010, *p* = 0.520] or dizygotic [rank-based ICC (*n* = 22) = −0.019, *p* = 0.535] twins. Similarly, bootstrap analyses did not reveal stark contrasts between sibling differences in the proportion of hits for either monozygotic (average absolute difference between twins |Δ| = 13.5%, *p* = 0.584) or dizygotic (|Δ| = 13.7%, *p* = 0.606) twins as compared to unrelated-infant differences (5th percentile |Δ| = 9.7%).

### Reaction time

We evaluated reaction times in a Gaussian mixed-effects model with fixed and random effects similar to other outcome variables, with the additional inclusion of a fixed effect for gaze-following accuracy (hit or miss). Reaction time was defined as the duration between the time beginning with the actress's gaze cue and the time the child made an eye movement toward either the left or right side target or non-target.

**Figure 5:**
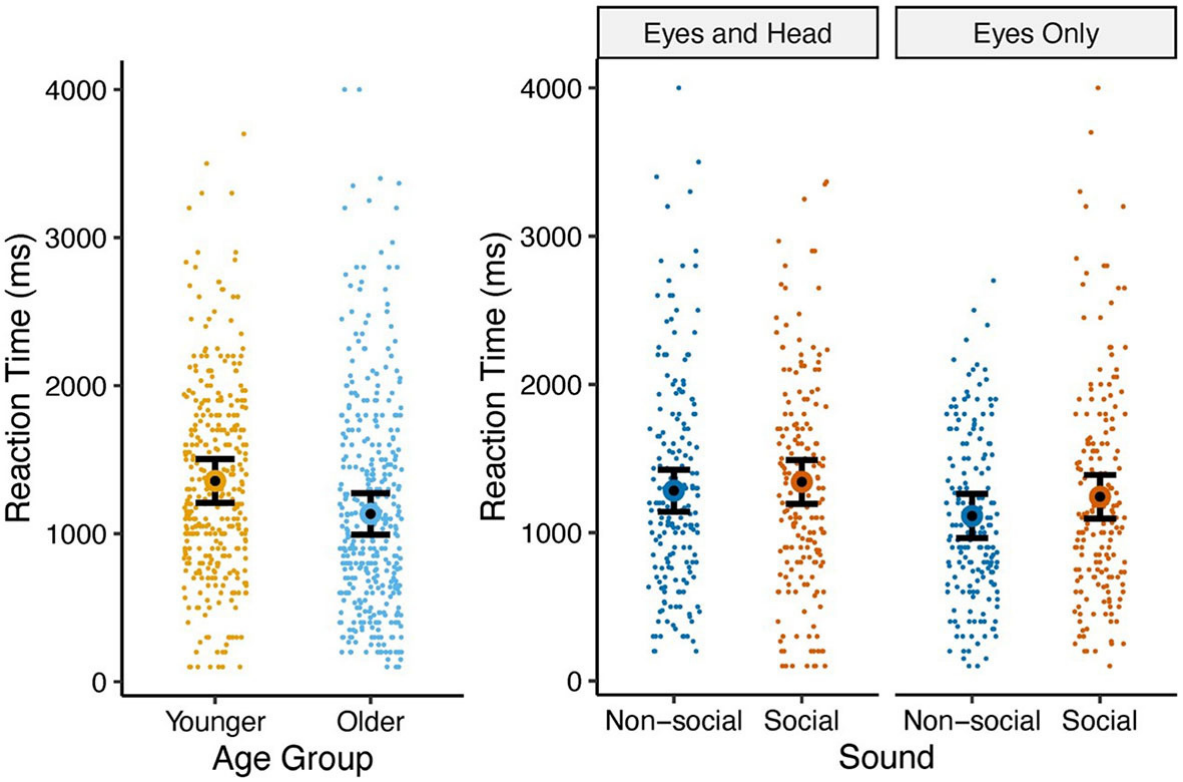
Means and standard errors of proportion of reaction times by age group **(left)** and gaze type × sound **(right)**. The reaction times represented in this graph were only those trials in which the participant achieved a hit.

ANOVA results (Table 5) showed the effects of age group such that older children had faster reaction times [1.13s (0.99s, 1.28s)] than younger children [1.36s (1.21s, 1.51s)] [*t*(55) = −2.165, *p* = 0.035]. Gaze type was also significant, with the Eyes Only condition eliciting quicker reaction times [1.18s (1.05s, 1.30s)] than the Eyes and Head condition [1.31s (1.19s, 1.43s)] [*t*(55) = −2.184, *p* = 0.033] (see Figure 5 for visualization). All other terms were non-significant.

**Table 5:** ANOVA of the reaction time.

**Variable**	**X^2^**	***p***
Age group	4.69	0.030^*^
Gender	0.27	0.603
Hit	0.21	0.643
Gaze type	4.77	0.029^*^
Pre-trial sound	2.33	0.127
Gaze type^*^sound	0.32	0.569

Outcome of the ANOVA for the model evaluating [Reaction Time ~ Age Group + Gender + Hit + Gaze Type ^*^ Pre-trial Sound + (1|Family/Child)].

**Figure 6:**
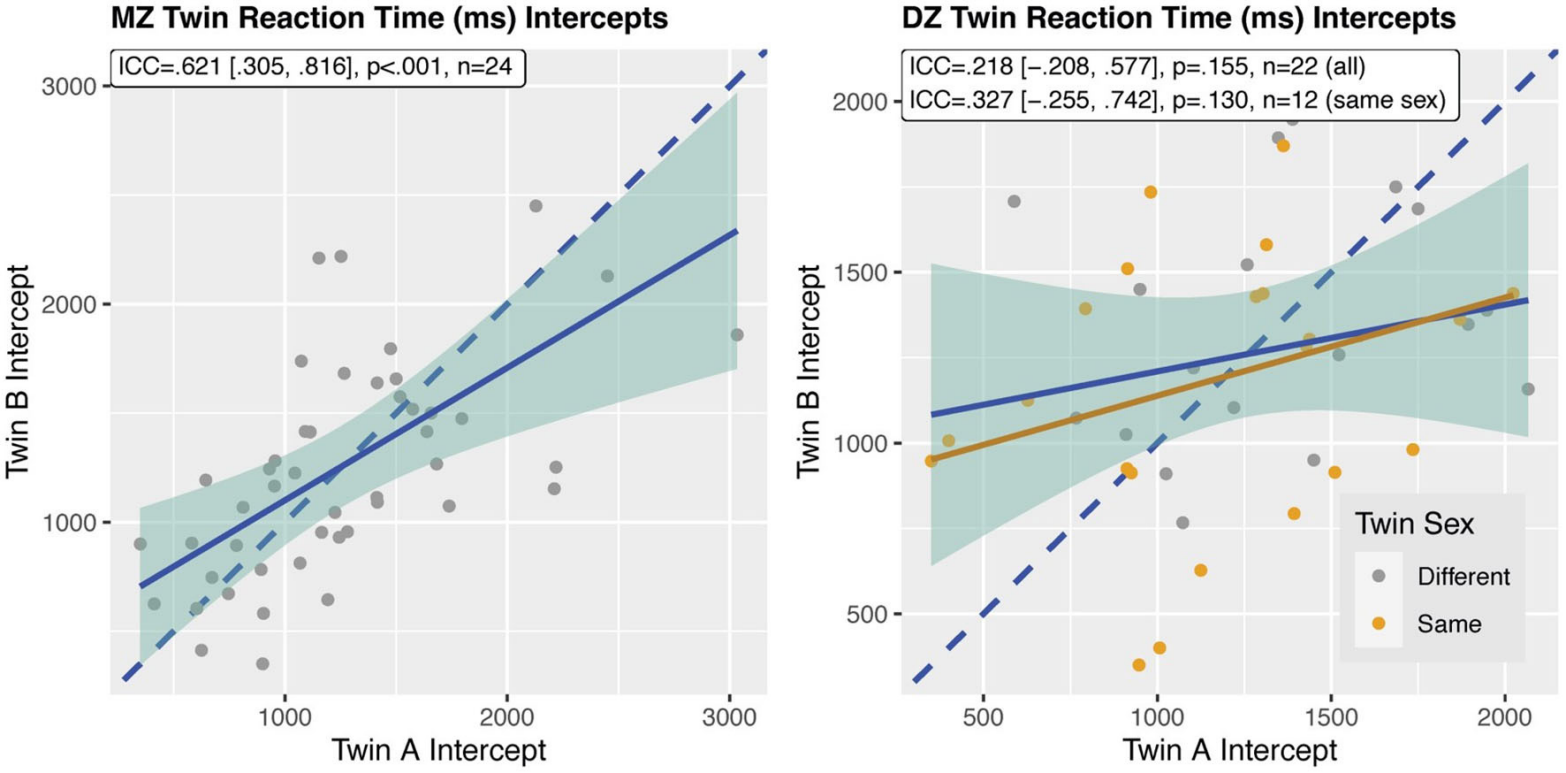
ICCs of reaction time between monozygotic (MZ, left) and dizygotic (DZ, right) twin pairs. ICCs for a smaller sample of same-sex twins are also provided. Solid blue line reflects linear fit of whole sample; solid yellow line reflects same sex DZ twin fit; dashed line is the line of perfect agreement between twins (slope m = 1).

Twin analyses showed that monozygotic twins had highly associated reaction times [ICC = 0.621 (0.305, 0.816), *p* <0.001; rank-based ICC = 0.552, p <0.001], but it also showed that dizygotic twins [ICC = 0.218 (−0.208, 0.577), *p* = 0.155], did not have correlated reaction times, even when only same-gender twins were considered [ICC = 0.327 (−0.255, 0.742), *p* = 0.130] (see Figure 6). Similarly, bootstrap analyses indicated sibling differences in reaction times for monozygotic (average absolute difference between twins |Δ| = 0.38s, *p* = 0.020) but not dizygotic (|Δ| = 0.45s, *p* = 0.119) twins, as compared to unrelated-infant differences (5th percentile |Δ| = 0.41s).

## Discussion

In this section, we have presented the scientific results, followed by more practical and technical aspects.

As hypothesized, we found a significant age effect for gaze following in infancy, which was highly expected given previous research (Moore, 2008; Del Bianco, 2019). Furthermore, we found an expected effect of visual cue type (Eyes and Head vs. Eyes Only), where the latter elicited less gaze following. This result, too, is in line with previous research (Moore and Corkum, 1998; Thorup et al., 2016; Nyström et al., 2019). Together, these results anchor our research against previous, well-known results from gaze-following research on infants, supporting methodological validity. It is notable that we modeled our visual manipulation on a recent live eye-tracking study (live interaction between the infant and experimenter), and, although we studied gaze following remotely via tablets using videos, we observed similar conditions and age effects (Thorup et al., 2016; Nyström et al., 2019). These findings showed that it is possible to study the central aspects of infant development remotely without any need for the involvement of the experimenter during data collection sessions.

In terms of the second manipulation—that of auditory cueing using communicative voice vs. Non-social Sound—we found no independent effect on gaze-following ability (the proportion of hits). This result relates to an ongoing discussion of whether gaze following is facilitated by special communicative cues (Senju and Csibra, 2008; Ishikawa and Itakura, 2019) or whether any type of alerting sound can bring about the same effect (Gredebäck et al., 2018). Our result is more in line with the latter view, but we acknowledge that we may be underpowered to detect a weak effect that may still be of theoretical relevance.

There are limited studies on the contribution of genes and environments to gaze behavior in infancy (but see Viktorsson et al., 2021). One recent study of a small sample of twins suggested very high heritability for eye vs. mouth looking and some other eye movement parameters in toddlerhood (Constantino et al., 2017). The pattern of intra-pair similarities (MZ vs. DZ) in the current study suggests that individual differences in gaze-following accuracy (hit vs. miss) do not reflect familial influences (shared genetics or environment). In contrast, we found that intra-pair similarity was very high in MZ twins for the latency to produce a gaze shift, while the DZ similarity was not significant. This pattern is consistent with the high heritability of this variable (Constantino et al., 2017). We did not find any strong relationship between reaction times and gaze-following accuracy, so this heritable trait likely reflects broader reactive impetus and/or oculomotor efficiency than gaze-following efficiency, *per se*. Similarly, it is important to note that, although we refer to these variables as reaction times in this article, eye movements are not triggered by peripheral events. Rather, they represent the time it takes from the central cue (the gaze shift of the model) to when the eyes of the infant move away from that cue. Thus, the attentional phenomenon we capture here is likely more complex than reflexive orienting to peripheral cues.

In terms of practical implications, a key question concerning data loss using the current remote approach as compared to in-laboratory studies. A prior study examining gaze following in infants using live in-laboratory probes (Thorup et al., 2016) averaged 78.8% trial retention across conditions for all participants who contributed enough data for analysis. This in-laboratory study included fewer trials (16 as compared to 32 in this study), which can lead to higher trial retention at some cost in experimental power (DeBolt et al., 2020). More critically, all trials in the live probe study were preceded by up to three attempts to ensure the infant's focus was centered on the visual cue, including using the baby's name and motherese between trials. In the current study, a substantial proportion of trials (17.3%) were lost due to the infant not looking at the actress's face prior to the gaze cue. Additional cueing in these situations to draw attention to the face explicitly would have likely increased trial retention. While the comparison is not exact, discounting trial loss due to gaze cue inattention, the current project demonstrates a reasonable level of data retention (60.2% + 17.3% = 77.5% total) as compared to the in-laboratory study (78.8% in Thorup et al., 2016).

This study used a remote, asynchronous testing approach relying on personal tablets (iPads) and showed that it is possible to obtain reasonable and scientifically significant data from infant (twin) families living across a large geographical area. Compared to prior comparable laboratory-based studies (Thorup et al., 2016), which were confined to a distinct geographic region, the ultimate sample in this study was much more geographically representative of the country as a whole, as evident from the study recruitment map (Supplementary Figure S1). These increases in geographic diversity were similarly noted in a significant proportion of developmental psychology experiments when transitioned to online platforms (Shore et al., 2023). However, as also noted by Shore et al. (2023), improvements in the overall sample diversity as facilitated by transitions to online studies are nuanced at best. In the current remote study, while the final sample showed a generally lower family salary as compared to the in-laboratory sample, the education levels between the two studies did not significantly differ. These findings suggest that, while remote delivery of infant studies on attention seems to readily surmount some geographic challenges, without additional outreach, they may still fall short of reaching families across broader dimensions of diversity. Even with the best-in-class protocols and technologies, some aspects of inclusivity may remain stubbornly out of reach. For example, in the country of the current study, examining race is complicated by cultural, scientific, and legislative prohibitions against the categorization of people by race due to pervasive beliefs regarding race as a social construct (Hübinette and Mählck, 2016; von Brömssen, 2021; Osanami Törngren, 2022). Nonetheless, while geography is not the only barrier to inclusivity, it is a significant component contributing to the isolation of populations stratified by demography in general. From this perspective, this research may highlight the optimistic potential of increased access while acknowledging significant challenges that are yet to be addressed.

Relatedly, it is important to discuss the low recruitment rate obtained (3.51%). One likely contributing factor is the fact that we required the families to have a specific type of tablet (iPad). Increasing the number of compatible systems would likely increase enrollment. However, even with this in mind, the recruitment seems surprisingly low, given that iPad coverage in Sweden is rather good (88.1% tablet market share in Sweden in 2019; StatCounter, 2023) as is the prevalence of tablet ownership in general, even discounting shared ownership by families (5.3 million tablet users in 2018 out of a total Swedish population of ~10 million; Thormundsson, 2022). In an in-laboratory study of infant twins that was conducted in the Stockholm area in parallel to this study, ~50% of the entire infant twin population expressed interest in participating in principle, and the final sample included 30% of the population (Falck-Ytter et al., 2021). While these families were specific to Stockholm, and the current population was specifically not from the same area, our team had believed it reasonable to assume that both populations would be similarly interested in contributing to research in general. Future research could investigate this assumption using community-based research. Similarly, twin studies of older children in Sweden obtained coverage of ~70% of the population (Anckarsäter et al., 2011), and interestingly, these studies were conducted remotely (but not asynchronously). In this study, we can only provide some speculations on possible reasons for the differences: (1) Despite our efforts to simplify the instructions, it is possible that some felt the procedures as too difficult to follow on their own, without more personal support; (2) it is possible that the remote *asynchronous* approach is too impersonal, making parents feel that they are not targeted by someone who really wants and needs *their* personal contributions; (3) it is possible that they did not feel comfortable with the handling of personal (video) data in the remote setting (transfer via the Internet, involvement of a site outside Sweden in data coding, etc.); and (4) it is possible that the reimbursement rate was too low. Future research should systematically address these and other potential factors to increase the coverage of remote studies because much of their value will depend on how many families can be enrolled.

That said, once families began to use the app, the transfer of their video data and completion of experimental sessions with their children were good. Viewability of videos, even with the remote provision of instructions and without direct communication by experimenters, was excellent, and the majority of submitted video trials were manually codable and analyzable. Robust data acquisition was supported by extensive testing and development of fail-safe algorithms mitigating the likelihood of data loss. Furthermore, the consideration of design features that allowed participant families control over their submitted materials may have improved participation confidence.

## Limitations of the study

The sample size is limited, and the results require additional reproduction. The use of manual coding is labor-intensive, and future work could consider computer-vision-based coding strategies (Chang et al., 2021; Li et al., 2022). That said, visible-light computer-vision-based eye tracking is limited in accuracy even with advanced calibration (Li et al., 2022) compared to modern infrared video oculographic techniques. In general, remote deployment is expected to result in greater heterogeneity and variability in environmental and experimental contexts than in laboratory settings where study confounds can be much more tightly controlled and where fewer tradeoffs may be needed to protect participant identities. For example, while instructions for adjusting volume were fairly clear (Supplementary Figure S3G), restrictions of the iPad operating system and a desire to minimize accidental receipt of additional personal information precluded direct measurement of application sound volumes or environment noise, which could have changed at any point during app usage. Beyond the relatively low family participation rate, this study and studies of infancy in general tend to target and recruit families of high socioeconomic status (SES) and thus do not reflect the diversity of the general population (Syed et al., 2018). Similarly, this study targeted Sweden alone and should be expanded to other countries, although the technical, legal, and potentially also ethical issues remain to be addressed.

## Data availability statement

The datasets presented in this article are not readily available because videos contain identifiable information about infants and parents. Requests to access the datasets should be directed to TF-Y (terje.falck-ytter@ki.se).

## Ethics statement

The studies involving humans were approved by the Regional Ethical Review Board in Stockholm. The studies were conducted in accordance with the local legislation and institutional requirements. Written informed consent for participation in this study was provided by the participants' legal guardians/next of kin. Written informed consent was obtained from the individual for the publication of any potentially identifiable images or data included in this article.

## Author contributions

FS and TF-Y were responsible for conceptualization, resources, project administration, and funding acquisition. TF-Y, PN, FS, KD, and JB contributed to the methodology. The software was designed and maintained by a combination of efforts from BL, JS, KD, FS, and TF-Y. Validation, formal analysis, investigation, and supervision were managed by TF-Y, FS, KD, and JB. Data were curated by FS, KD, JS, and TF-Y. The original manuscript was written by FS, TF-Y, and KD and was reviewed by all authors. FS and KD contributed to the manuscript visualization. All authors contributed to the article and approved the submitted version.
