# Supplementary material for: Remote, tablet-based assessment of gaze following: a nationwide infant twin study
Source: Front Psychol. 2023 Oct 3;14:1223267. doi: 10.3389/fpsyg.2023.1223267 (PMC10579944; doi:10.3389/fpsyg.2023.1223267)
Supplement: Supplementary file 1 [file Data_Sheet_1.PDF]

SUPPLEMENTARY MATERIAL

Supplemental Results – Age as a covariate

With consideration that early developmental phenomena associated with gaze following behaviors are unlikely to be completely linear with age, we chose a relatively simple strategy for grouping infants into categories of either Younger or Older based on their age in relation to a cutoff pegged to the median age of our sample. This approach made fewer assumptions regarding the joint relationship of infant age and targeted dependent variables, save for a hypothesis of monotonicity, while optimizing power for direct age comparisons. We had also considered other approaches, including the inclusion of age as a continuous linear variable rather than a categorical variable, however the relatively large overall age range of our infant sample was concerning given the potential for leverage effects at the tails of age. For completeness and transparency, we provide parallel analyses to that of the main text with age group replaced by continuous age in months. Of 7 significant statistical findings in age group based analyses, 6 were replicated when age group was replaced with age as a covariate. Age relationships with proportion of invalid data did not replicate, suggesting potential non-linear effects or a questionable strength of the original finding.

Proportion of Invalid Trials

ANOVA indicated main effects and gaze cue type and a gaze cue type x pre-trial sound interaction, but no other effects (Table S1). The Eyes Only condition led to more invalid trials (38.1% [31.7%, 45.0%]) than the Eyes and Head condition (32.9% [27.0%, 39.5%]) ($t(55) = 2.74$, $p=.008$). The interaction effect of gaze type and pre-trial sound was driven by the lowest proportion of invalid trials occurring in the Eyes and Head + Non-social Sound condition (29.7% [23.7%, 36.5%]; cf. Eyes and Head + Social Sound (36.4% [29.6%, 43.7%]), Eyes Only + Non-social Sound (38.7% [31.7%, 46.2%]), and Eyes Only + Social Sound (37.5% [30.6%, 44.9%]). *As compared to group-based age analyses, the effect of age was inconsistent, but all other detected effects were similar.*

Table S1. ANOVA of Proportion of Invalid Trials (Compare with Table 2)

Variable	X^2	P
**Age (Continuous)**	1.42	.234
**Sex**	0.004	.949
**Gaze Type**	7.53	.006 **
**Pre-trial Sound**	2.32	.128
**Gaze Type*Sound**	4.69	.030 *

*Note: Outcome of the ANOVA for the model evaluating [Proportion of Invalid Trials ~ Age + Sex + Gaze Type * Pre-trial Sound + (1|Family/Child)].*

Hits versus Misses

ANOVA indicated main effects of age group and gaze cue type with all other terms being non-significant (Table S2). Children had a higher probability of a hit as they got older ($p=.010$). The Eyes Only Condition elicited a lower probability of a hit (51.7% [48.4%, 54.9%]) than the Eyes

and Head condition (60.4% [57.3%, 63.4%]) (t(55) = -3.91, p<.001). All other terms were non-significant. *As compared to group-based age analyses, all detected effects were similar.*

Table S2. ANOVA of Proportion of Hits (Compare with Table 4)

Variable	X^2	P
**Age (Continuous)**	6.63	.010 **
**Sex**	0.04	.839
**Gaze Type**	15.3	<.001 ***
**Pre-trial Sound**	0.63	.428
**Gaze Type*Sound**	0.15	.697

*Note: Outcome of the ANOVA for the model evaluating [Proportion of Hits ~ Age + Sex + Gaze Type * Pre-trial Sound + (1|Family/Child)].*

Reaction Time

ANOVA results (Table S3) showed effects of age group such that older children had faster reaction times than younger children (p=.006). Gaze type was also significant, with the Eyes Only condition eliciting quicker reaction times (1.17s [1.05s, 1.29s]) than the Eyes and Head condition (1.31s [1.19s, 1.42s]) (t(55) = 2.174, p=.034). All other terms were non-significant. *As compared to group-based age analyses, all detected effects were similar.*

Table S3. ANOVA of Reaction Time (Compare with Table 5)

Variable	X^2	p
**Age (Continuous)**	7.70	.006 **
**Sex**	0.40	.527
**Hit**	0.19	.663
**Gaze Type**	4.73	.029 *
**Pre-trial Sound**	2.28	.131
**Gaze Type*Sound**	0.31	.577

*Note: Outcome of the ANOVA for the model evaluating [Reaction Time ~ Age Group + Sex + Hit + Gaze Type * Pre-trial Sound + (1|Family/Child)].*

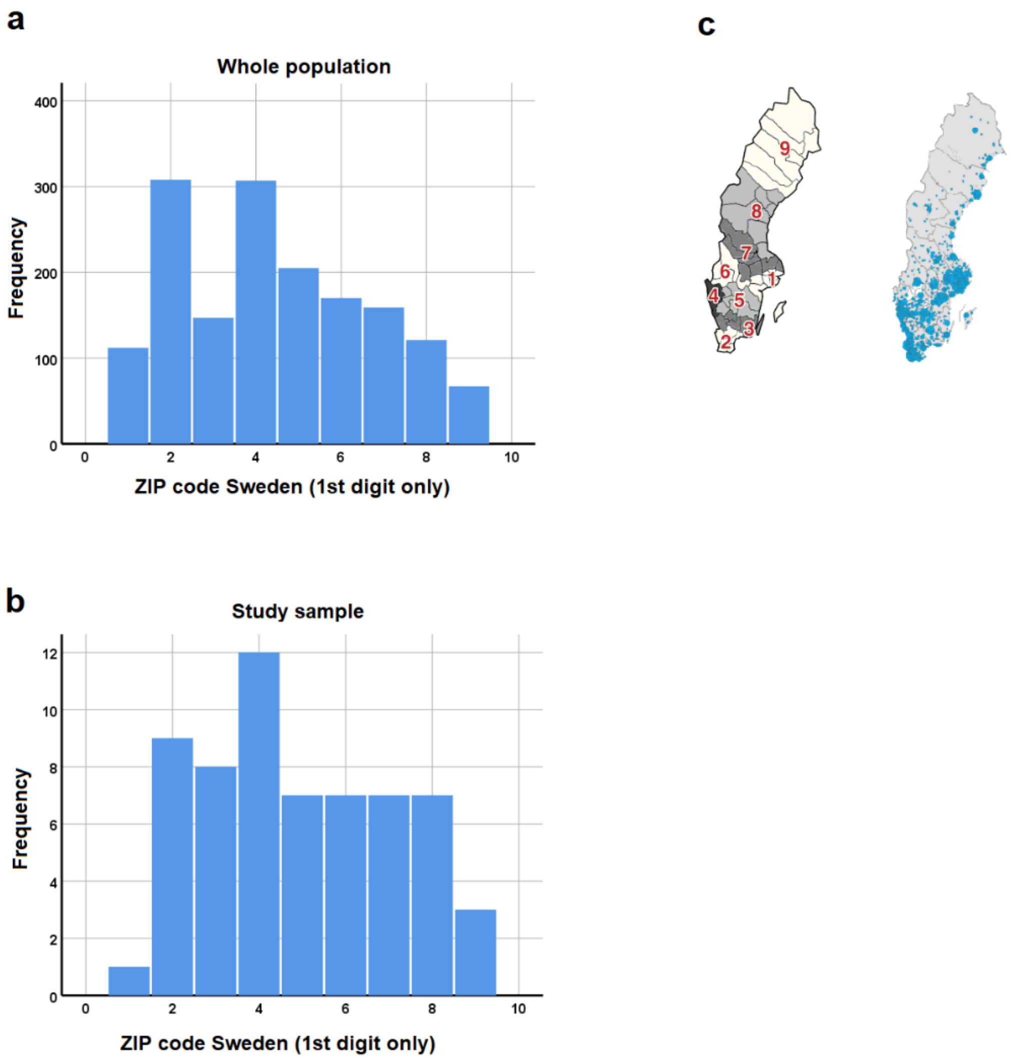

Figure S1. Geographical spread of study sample a) The study targeted all twins born in Sweden in a restricted age span and during a restricted time period, except families living in the greater Stockholm area (postal code starting with digit 1, hence this postal code is underrepresented; see main text for further information). The histogram shows the distribution in initial postal code digit of all families who received the letter. Peaks around 2 and 4 represent the cities Malmö and Gothenburg, respectively. **b**) As can be seen, the study sample mimicked the targeted population in terms of initial post code digit rather well. The study sample came across all over Sweden, including the less populated northern areas. **c**) map of the postal codes (1st digit only), together with population density map of Sweden. The latter is taken from Statistics Sweden, 2019.

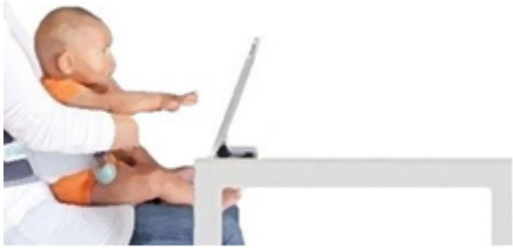

Figure S2. Figure from our recruitment materials, which included instructions on how to place the infant in relation to the tablet.

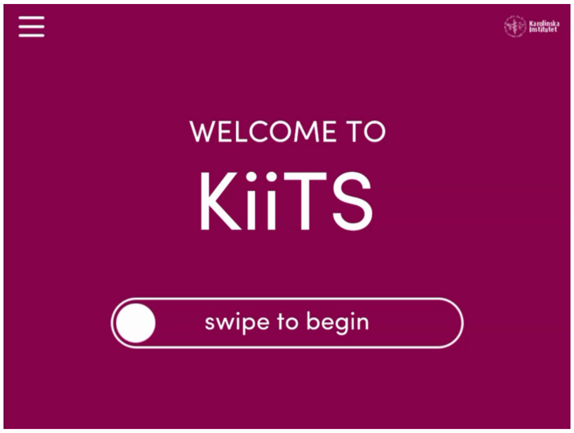

Figure S3a. App screenshot: Welcome

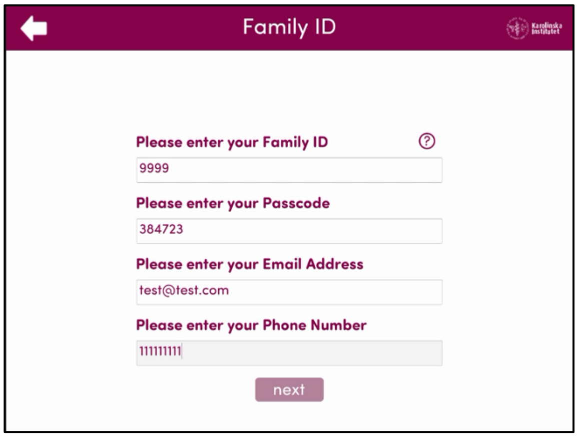

Figure S3b. App: family login

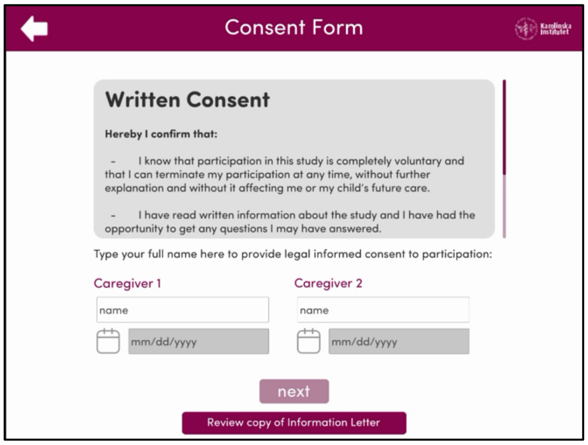

Figure S3c. App: consent

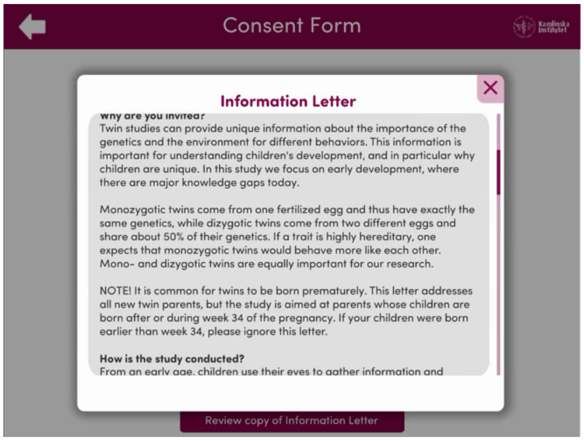

Figure S3d. App: Information Letter (optional – also in mailed invitation letter)

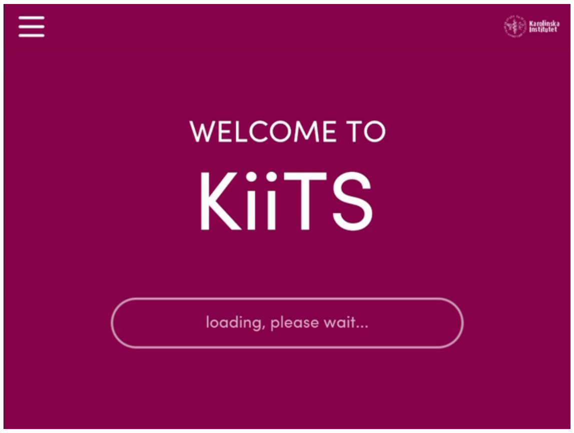

Figure S3e. App: Loading page

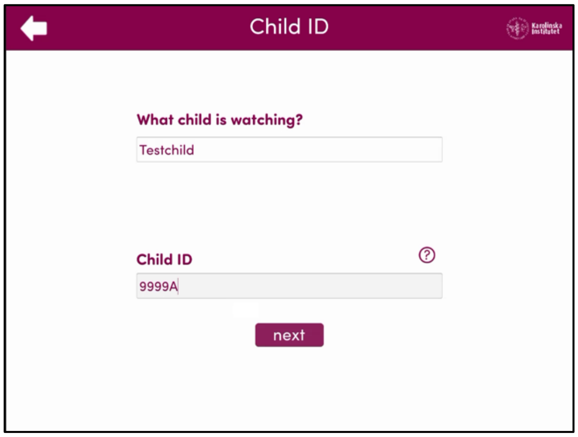

Figure S3f. App: Child selection

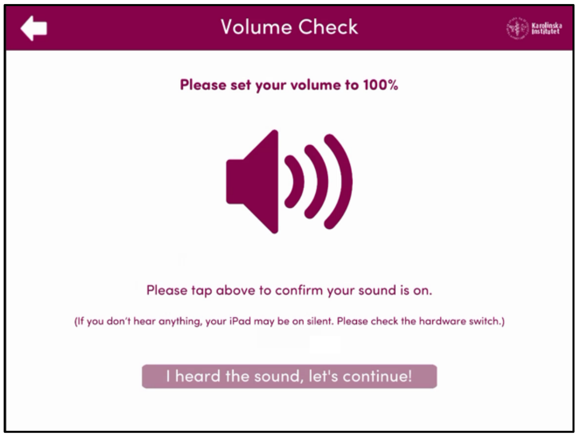

Figure S3g. App: Volume adjustment/ verification

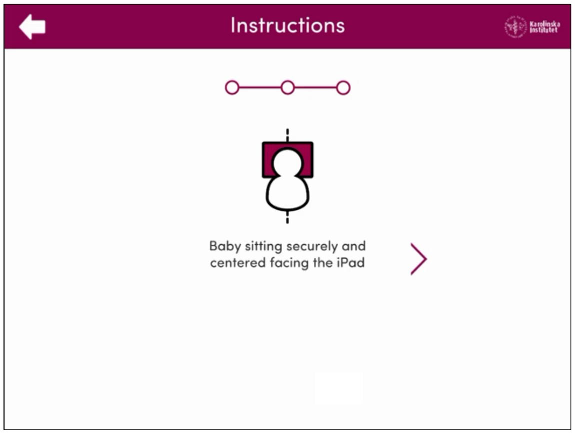

Figure S3h. App: Instructions – Infant Position

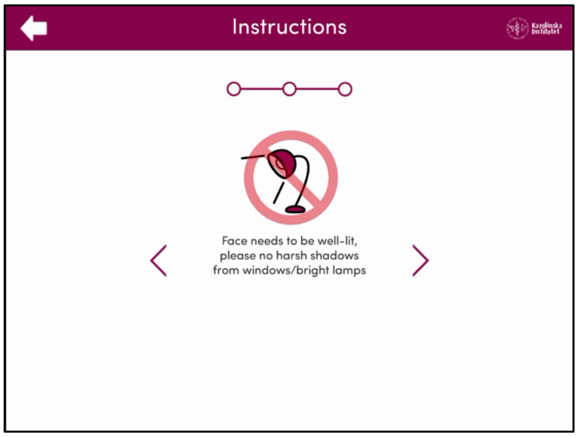

Figure S3i. App: Instructions - Room lighting guide

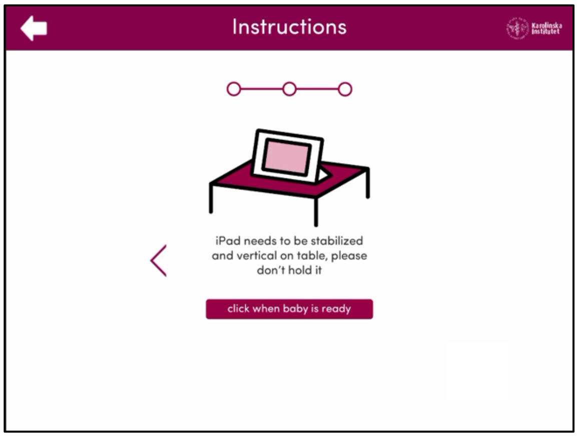

Figure S3j. App: Instructions – Tablet position

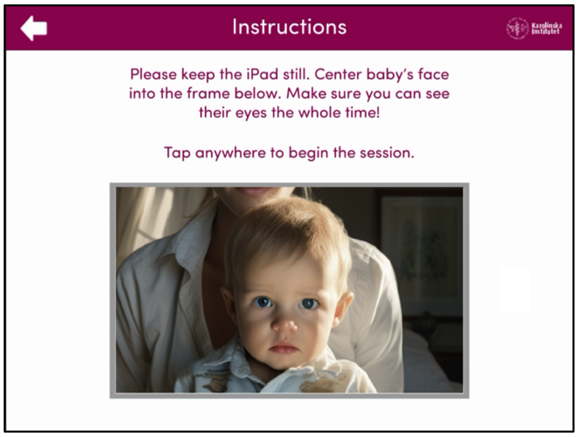

Figure S3k. App: Positioning live stream. Followed by experiment, then back to Welcome (**Figure S3a**). *Infant face AI generated by Midjourney.*

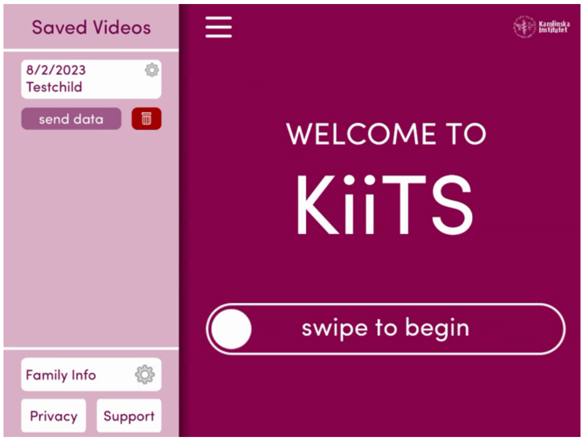

Figure S3l. App: Video upload menu

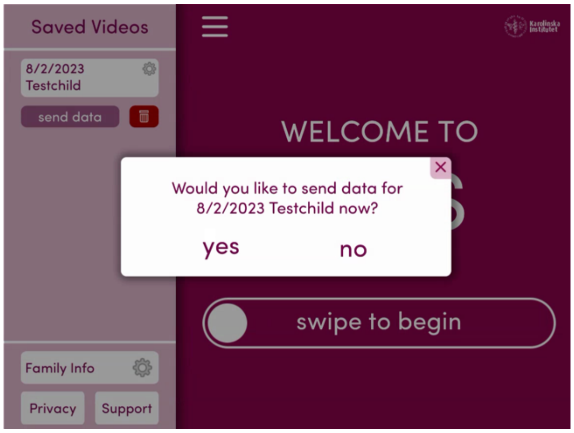

Figure S3m. App: Video upload confirmation screen.

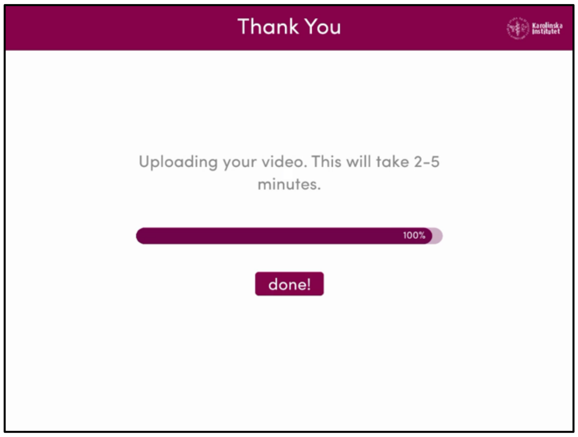

Figure S3n. App: Video upload progress
